# Endoscopic diagnosis and treatment of colon cancer: current evidence and future directions

**DOI:** 10.3389/fsurg.2026.1816264

**Published:** 2026-09-01

**Authors:** Huimei Gong, Hongyu Li, Zhiwen Zheng, Hanbin Zhu, Yanni Zhang

**Affiliations:** 1 Department of Gastroenterology, People’s Hospital of Lincang City, Lincang, China; 2 Department of Gastroenterology, Changxing County People’s Hospital, Huzhou, Zhejiang, China; 3 Department of Gastroenterology, Changhai Hospital, Naval Medical University, Shanghai, China

**Keywords:** artificial intelligence, colon cancer, early colon cancer, endoscopic full-thickness resection, endoscopic mucosal resection, endoscopic submucosal dissection, endoscopic ultrasonography

## Abstract

Colorectal cancer remains a major cause of cancer morbidity and mortality, while screening and advances in endoscopic imaging have increased detection of lesions that may be amenable to organ-preserving treatment. Accurate assessment of morphology, optical pattern, and invasion depth is central to selecting among endoscopic mucosal resection (EMR), endoscopic submucosal dissection (ESD), endoscopic full-thickness resection (EFTR), and surgery. This structured narrative review synthesizes contemporary evidence and major society guidance on endoscopic diagnosis and treatment of early colon cancer, with particular attention to comparative effectiveness, evidence limitations, regional practice differences, surveillance, recurrence management, patient-centered decision-making, and implementation of artificial intelligence. EMR remains the principal approach for most large low-risk nonpedunculated lesions because of procedural efficiency and favorable safety, although piecemeal resection limits histologic staging and historically carries a higher risk of local recurrence. ESD provides *en bloc* resection and more reliable pathologic assessment for lesions with suspected superficial submucosal invasion or other features requiring precise staging, at the cost of longer procedures, greater technical demands, and higher perforation risk. EFTR has a complementary role for selected small non-lifting, fibrotic, or difficult-location lesions, but long-term oncologic evidence is less mature. Cross-study comparisons are limited by heterogeneous lesion selection, operator expertise, outcome definitions, and follow-up. Major European, Japanese, and US recommendations share a risk-based approach but differ in the practical positioning of ESD, reflecting regional expertise and referral infrastructure. Artificial intelligence improves adenoma detection in randomized trials, yet evidence for early cancer detection, invasion-depth prediction, interval cancer prevention, and survival remains insufficient for uncritical implementation. A structured, multidisciplinary approach that integrates lesion biology, local expertise, pathology, patient preferences, and the possibility of additional surgery after non-curative resection is essential.

## Introduction

Colorectal cancer remains a major global health burden, with more than 1.9 million new cases and more than 900,000 deaths estimated in 2020. The burden is projected to increase substantially by 2040 as populations age and incidence rises in many transitioning countries (1, 2). Screening programs and improved colonoscopy have shifted a proportion of diagnoses toward earlier, potentially curable disease, creating a larger role for minimally invasive organ-preserving treatment (3, 4).

For the purposes of this review, early colon cancer refers to carcinoma confined to the mucosa or submucosa. The clinical challenge is not simply to identify T1 disease, but to distinguish lesions with a sufficiently low probability of lymph node metastasis to justify local treatment from those requiring oncologic resection with lymphadenectomy. Histologic differentiation, lymphovascular invasion, tumor budding, and other adverse features influence nodal risk; importantly, recent evidence questions the use of deep submucosal invasion as an isolated determinant when other adverse features are absent (5, 6). This uncertainty reinforces the need to integrate optical diagnosis, resection quality, pathology, and patient factors rather than rely on a single threshold.

High-definition white-light endoscopy, image-enhanced endoscopy, magnification, and pit-pattern assessment have improved real-time estimation of invasion depth (7–10). At the same time, EMR, ESD, and EFTR have expanded the range of lesions that can be managed without formal colectomy. These techniques are not interchangeable. They differ in their ability to achieve *en bloc* and R0 resection, obtain an intact specimen for staging, manage fibrosis, prevent local recurrence, and avoid adverse events. They also differ markedly in training requirements and availability.

The aim of this review is therefore not to propose a new classification or claim conceptual novelty, but to provide an updated clinically oriented synthesis that links diagnosis, lesion risk stratification, selection among EMR, ESD, EFTR, and surgery, post-resection pathology, surveillance, guideline differences, and emerging technologies. Particular emphasis is placed on quantitative outcomes, limitations of the underlying evidence, controversies relevant to Western and Asian practice, and patient-centered decision-making.

## Methods

### Review design and search strategy

This article was prepared as a structured narrative review. Targeted searches were conducted in PubMed/MEDLINE and on the official websites or publication platforms of major gastrointestinal endoscopy societies. The principal search period was January 1, 2000 through June 30, 2026. Older landmark studies were retained when they established foundational optical classifications, histopathologic risk concepts, or other evidence that remains directly relevant to current practice.

Search concepts were combined using terms related to colon or colorectal cancer, early cancer, T1 cancer, large nonpedunculated colorectal lesions, laterally spreading tumor, optical diagnosis, narrow-band imaging, JNET, NICE, pit pattern, endoscopic ultrasonography, EMR, underwater EMR, cold EMR, ESD, traction-assisted ESD, EFTR, full-thickness resection device, recurrence, surveillance, lymph node metastasis, artificial intelligence, computer-aided detection, computer-aided diagnosis, quality of life, shared decision-making, and guideline. Reference lists of high-level reviews and major guidelines were also screened to identify relevant primary studies and landmark evidence.

### Evidence selection and prioritization

Evidence was selected for direct relevance to early colon cancer or to colorectal lesions when colon-specific evidence was limited. Priority was given to contemporary society guidelines, randomized controlled trials, systematic reviews and meta-analyses, prospective multicenter studies, large registries, and large cohort studies. Smaller retrospective series were used selectively for uncommon indications, technical innovations, or emerging areas in which higher-level evidence was unavailable. Studies focused exclusively on rectal cancer were generally not used to support colon-specific recommendations unless the underlying diagnostic or endoscopic principle was explicitly applicable to colorectal lesions.

### Evidence appraisal and synthesis

Because this is a structured narrative review rather than a systematic review, no *de novo* meta-analysis was performed and formal GRADE ratings were not assigned. Evidence was appraised according to study design, consistency across studies, directness to the clinical question, applicability across practice settings, duration and completeness of follow-up, and major sources of bias. Particular attention was paid to selection and referral bias, confounding by lesion morphology and size, operator and center volume, regional expertise, prior manipulation of lesions, inconsistent definitions of technical success and curative resection, and incomplete adjustment in nonrandomized comparisons. Quantitative outcome ranges are presented to support clinical interpretation, but they should not be read as pooled estimates or as valid head-to-head comparisons when case mix differs substantially.

During manuscript revision, ChatGPT (GPT-5.5 Thinking model, version accessed July 2026; OpenAI, https://openai.com/) was used for drafting support, language editing, structural refinement, and consistency checking. All scientific content, factual claims, citations, and references were reviewed and verified by the authors, who take full responsibility for the final manuscript.

## Endoscopic assessment and risk stratification of colonic lesions

Treatment selection begins with careful lesion assessment before biopsy, tattooing, or incomplete resection alters the submucosal plane. Morphology, location, surface and vascular pattern, focal depression or nodularity, ulceration, and prior manipulation should be documented. Laterally spreading tumors are heterogeneous: homogeneous granular lesions generally have lower risk of submucosal invasion, whereas non-granular lesions and granular mixed lesions with a dominant nodule or depression carry greater risk and more often require *en bloc* staging (7, 8).

Image-enhanced endoscopy should be interpreted through standardized systems rather than isolated visual impressions. The JNET classification links vascular and surface patterns to a spectrum from low-grade neoplasia to deep invasion, although type 2B remains a clinically challenging intermediate category (9). Magnifying chromoendoscopy with Kudo pit-pattern assessment remains valuable when available; destructive or non-structured type V patterns increase concern for deep invasion (10). In routine practice, diagnostic performance is influenced by expertise and image quality, and no single classification eliminates understaging or overstaging.

The non-lifting sign requires context. A firm non-lifting lesion with an invasive optical pattern should raise concern for deep invasion and generally prompt surgical assessment. In contrast, non-lifting caused by prior biopsy, tattooing, inflammation, or incomplete resection may reflect fibrosis rather than cancer and can remain amenable to ESD or EFTR in expert centers (11–14). Repeated unsuccessful snare attempts should be avoided because additional fibrosis can narrow subsequent treatment options.

## Advanced endoscopic imaging and detection

Electronic chromoendoscopy improves visualization of mucosal and vascular detail without dye spraying. Narrow-band imaging and related contrast-enhancement platforms are particularly useful for characterization after a lesion is detected, whereas linked color imaging can improve lesion conspicuity. Their value depends on standardized interpretation, adequate bowel preparation, stable visualization, and operator training (15, 16).

Confocal laser endomicroscopy can provide microscopic *in vivo* imaging and has been studied as an optical biopsy technique, but routine use in early colon cancer remains limited by cost, procedural complexity, contrast requirements in many protocols, and the continuing need for definitive histopathology after curative-intent resection (17). Accordingly, it is best viewed as a selective adjunct rather than a replacement for tissue diagnosis.

A single universal early cancer detection rate is not meaningful because detection depends on population risk, colonoscopy indication, bowel preparation, withdrawal technique, endoscopist performance, and lesion morphology. Flat, depressed, right-sided, fold-hidden, and subtly serrated lesions remain important sources of miss. Quality programs therefore rely on established colonoscopy quality metrics rather than early cancer detection rate alone.

## Role of endoscopic ultrasonography in colon cancer

Cross-sectional imaging remains central when advanced invasion, nodal disease, or distant metastasis is suspected. EUS can visualize bowel-wall layers and may provide additional information on local invasion depth, but its role proximal to the rectum is constrained by access, operator dependence, fibrosis-related overstaging, and variable accuracy. A systematic review and meta-analysis found useful but imperfect performance for T staging of colonic cancer proximal to the rectum, and comparative data do not establish EUS as a replacement for high-quality optical diagnosis or computed tomography (18, 19).

In practice, EUS is most defensible as a selective problem-solving tool when optical findings are equivocal and the result could change management. It should not be used as a stand-alone method for nodal staging. The limited and heterogeneous evidence base also means that routine EUS before endoscopic resection of an apparently superficial colonic lesion cannot be recommended.

Table 1 summarizes the evidence synthesis from available systematic and comparative studies rather than a stand-alone guideline recommendation (18, 19).

**Table 1:** Role of endoscopic ultrasonography in early colon cancer.

Clinical question	Potential contribution	Major limitations	Practical interpretation
Depth of invasion	Layer-by-layer assessment may help distinguish superficial from deeper invasion in selected cases.	Access, fibrosis, inflammation, lesion location, and operator dependence can reduce accuracy.	Adjunct when optical assessment is inconclusive; not routine for all lesions.
Precise T substaging	May contribute anatomic detail in selected lesions.	Performance is less reliable for exact substaging than for broad superficial versus advanced separation.	Do not allow a marginal EUS distinction alone to override the total clinical picture.
Nodal staging	May visualize nearby nodes.	Morphologic and size criteria are insufficiently accurate for independent decision-making.	Use cross-sectional staging and multidisciplinary assessment.
Comparison with optical diagnosis	Can provide complementary information.	Does not consistently outperform modern image-enhanced optical assessment.	Optical diagnosis remains the primary endoscopic triage tool.
Evidence maturity	Systematic review and comparative cohort evidence are available.	Small studies, heterogeneity, and limited proximal-colon data remain important.	Interpret selectively and avoid routine use claims.

## Endoscopic mucosal resection

### Indications and technique

EMR remains the standard endoscopic approach for most large nonpedunculated adenomatous lesions without features of deep invasion. The 2024 ESGE guideline recommends conventional diathermy-based EMR for large lesions of 20 mm or greater and emphasizes *en bloc* techniques when superficial invasive carcinoma is suspected and standard snare resection cannot provide adequate staging (11). US and Japanese guidance similarly support lesion characterization before resection and referral of complex lesions to appropriately experienced endoscopists (13, 14).

Conventional EMR uses submucosal injection to separate the lesion from the muscularis propria, followed by snare resection. The aim is complete removal in the fewest safe pieces. Underwater EMR can facilitate capture in selected lesions by altering mucosal configuration without a conventional lift. Cold snare resection has attracted interest because it avoids deep thermal injury, but the balance between safety and residual or recurrent tissue remains lesion dependent.

### Outcomes and evidence limitations

For lesions larger than 20 mm, piecemeal resection is common and limits lateral-margin assessment. Historical recurrence after wide-field piecemeal EMR has often been in the 10% to 20% range, although expert surveillance and contemporary recurrence-prevention strategies substantially improve outcomes (20, 21). Randomized evidence also demonstrates that technical modifications can alter the safety and efficacy profile of large-polyp resection, underscoring that the term EMR does not describe a single uniform intervention (22).

Margin thermal ablation after complete visible resection is one of the clearest advances in modern EMR. Randomized evidence shows that thermal ablation of the post-EMR margin reduces adenoma recurrence (21). This improvement is central to the current EMR-versus-ESD debate because older recurrence estimates can overstate the recurrence expected after high-quality contemporary EMR.

The principal evidence limitation is selection. EMR cohorts predominantly include lesions believed to be low risk for invasive cancer, while lesions requiring intact histologic staging are preferentially referred for ESD or surgery. Apparent differences in recurrence, adverse events, or oncologic outcomes therefore cannot be interpreted without considering baseline lesion biology and the reason a technique was selected.

### Adverse events and surveillance

Major EMR adverse events include intraprocedural bleeding, delayed bleeding, deep mural injury, perforation, and post-polypectomy syndrome. Prevention depends on stable access, appropriate injectate and electrosurgical technique, recognition of deep mural injury, selective defect closure, and prompt treatment of visible vessels. Large right-sided defects deserve particular attention because delayed bleeding risk is greater.

After piecemeal resection of a large lesion, early scar assessment is essential. US consensus guidance recommends a short-interval examination after piecemeal resection of lesions 20 mm or larger, and subsequent intervals depend on scar findings, histology, and the broader post-polypectomy context (23). Residual or recurrent adenoma is frequently treatable endoscopically when detected early.

## Endoscopic submucosal dissection

### Indications and technical considerations

ESD enables *en bloc* resection independent of snare size and is most valuable when an intact specimen is needed to determine whether endoscopic treatment is curative. Major indications include lesions with suspected superficial submucosal invasion, non-granular morphology, focal depression, fibrosis or non-lifting without clear deep invasion, and selected recurrent lesions. ESGE recommends selective use in lesions requiring *en bloc* resection and in high-volume centers, while Japanese guidance positions ESD more broadly within an established training and referral infrastructure (12, 14).

Colorectal ESD is technically demanding because of the thin wall, colonic mobility, narrow submucosal plane, paradoxical scope movement, and fibrosis after prior manipulation. Safe performance requires controlled mucosal incision, repeated maintenance of the submucosal cushion, precise dissection above the muscularis propria, hemostasis, and immediate management of muscle injury. Procedure planning should account for lesion location, expected fibrosis, operator experience, rescue closure capability, and access to surgical support.

Traction-assisted approaches improve exposure of the submucosal plane and can reduce technical difficulty. Evidence across traction methods supports better visualization and, in some settings, improved procedural efficiency, although devices and operator familiarity vary (24).

### Outcomes and evidence limitations

A systematic review and meta-analysis reported *en bloc* resection around 90% and R0 resection around the low-80% range overall, with important regional differences and heterogeneity (25). In expert series, *en bloc* rates frequently exceed 90% and R0 rates commonly fall around 80% to 90%. After curative *en bloc* resection, local recurrence is generally very low, often below 2%. The tradeoff is longer procedure time and a higher perforation risk than standard EMR, commonly reported in the low single digits to approximately 8% depending on case mix and expertise.

The evidence base is strengthened by large series, systematic reviews, and increasing prospective data, but it remains vulnerable to center-volume effects and referral bias. Outcomes from expert Asian centers cannot be assumed to transfer unchanged to low-volume settings. Conversely, poorer early Western results may not represent outcomes achievable after structured training and centralization. Recent prospective evidence continues to mature the long-term oncologic assessment of ESD for T1 disease (26).

### Adverse events

Perforation and delayed bleeding are the principal major adverse events. Prevention centers on staying in the correct plane, traction when needed, prophylactic treatment of significant vessels, avoidance of excessive thermal injury, and immediate closure of recognized perforation or deep muscle injury. Most small intraprocedural perforations can be managed endoscopically in stable patients, whereas delayed perforation, uncontrolled bleeding, or peritonitis requires urgent surgical evaluation.

## Comparative evidence: EMR versus ESD

EMR and ESD should be considered complementary rather than universally competing techniques. Current European and US guidance generally favors EMR for most large low-risk nonpedunculated lesions, whereas ESD is reserved for lesions in which *en bloc* histology is important or standard EMR is unlikely to achieve an oncologically adequate specimen (11–13). Japanese guidance places greater emphasis on ESD in appropriately selected lesions within a practice environment with broader expertise (14).

Quantitatively, contemporary evidence consistently shows higher *en bloc* and R0 resection with ESD. Meta-analytic ESD estimates are approximately 90% for *en bloc* resection and about 80% to 90% for R0 resection, while *en bloc* EMR becomes progressively less feasible as lesion size exceeds 20 mm (25). Historical recurrence after piecemeal EMR is often about 10% to 20%, whereas recurrence after curative *en bloc* ESD is usually below 2%; however, modern margin thermal ablation can reduce EMR recurrence to low single-digit levels in high-quality practice (20, 21). ESD generally carries longer procedure times and a higher perforation risk, while EMR has a lower technical burden and wider availability.

These figures should not be treated as direct comparative effect estimates. ESD populations are enriched for lesions with fibrosis, non-granular morphology, suspected superficial invasion, or other complex features, whereas EMR populations are enriched for lesions believed to be noninvasive. Studies also differ in definitions of R0 and curative resection, pathology processing, surveillance intensity, endoscopist experience, and access to rescue surgery. Therefore, the clinically relevant question is not which technique has the best unadjusted outcome, but which technique provides adequate staging and durable control for a specific lesion in a specific practice setting.

For carefully selected low-risk T1 colorectal cancer, meta-analysis suggests that primary endoscopic resection can achieve long-term outcomes comparable to surgery in appropriately selected patients, while adverse histology requires consideration of additional oncologic surgery (27). This comparison remains subject to selection bias because healthier patients and higher-risk tumors are more likely to undergo surgery.

## Endoscopic full-thickness resection

### Clinical role and indications

EFTR occupies a narrower, complementary position. The clip-assisted full-thickness resection device is most useful for selected small lesions that are non-lifting because of fibrosis, recurrent after prior resection, or situated in locations where conventional EMR is unlikely to succeed and ESD may be disproportionately difficult. The technique should not be framed as a replacement for colectomy in lesions with clear deep invasion or a substantial risk of nodal disease.

Multicenter data support technical feasibility for difficult colonic lesions, but device capacity constrains lesion size and the ability to capture fibrotic tissue. Outcomes are generally best for lesions around 20 mm or smaller, although practice varies (28). Lesions at the appendiceal orifice require special caution because post-procedure appendicitis is a clinically important risk (29).

### Outcomes and evidence limitations

Across prospective and multicenter series, technical success is commonly around 85% to 95% and R0 resection about 70% to 85%, with variation by lesion size, fibrosis, location, prior treatment, and operator experience (28). A single-center comparison of EFTR and ESD for selected lesions 30 mm or smaller illustrates the possibility of different technical tradeoffs, but nonrandomized comparisons remain highly confounded by indication and center expertise (30). Long-term oncologic evidence for early cancer is substantially less mature than for EMR and ESD.

Adverse events include post-procedural pain, bleeding, localized peritonitis, failure of secure closure, and site-specific complications. Because full-thickness wall capture is intentional, procedural safety depends on correct clip deployment before resection and careful post-procedure monitoring. The need for urgent surgical rescue must be anticipated rather than regarded as a remote exception.

Table 2 summarizes this practical framework by integrating optical diagnosis evidence and contemporary guideline principles (7–14).

**Table 2:** Evidence-based endoscopic features for risk stratification and treatment selection in colonic lesions.

Endoscopic feature	Key finding	Relative invasion concern	Preferred management framework
Granular LST, homogeneous	Uniform granular surface without depression	Low	EMR is usually appropriate; piecemeal resection acceptable when intact cancer staging is not required.
Granular LST, mixed type	Dominant nodule or focal depression	Intermediate	Targeted expert reassessment; *en bloc* resection preferred when superficial invasion is suspected.
Non-granular LST	Flat surface, pseudo-depression, subtle margins	Higher	ESD in expert centers for suspected superficial invasion; surgery for convincing deep invasion.
JNET type 2A	Regular surface and vessels	Low	EMR usually appropriate according to size and morphology.
JNET type 2B	Irregular surface or vessels	Intermediate	Expert reassessment; *en bloc* histology often preferred because deep invasion cannot be excluded reliably.
JNET type 3	Avascular or highly irregular pattern	High	Surgical assessment for likely deep invasion.
Pit pattern VI, mild irregularity	Irregular but not destructive pattern	Low to intermediate	En bloc resection when accurate staging is required.
Pit pattern VN	Non-structured or destructive pattern	High	Surgical assessment for likely deep invasion.
Non-lifting with fibrosis	Prior biopsy, tattoo, inflammation, or recurrence without invasive optical features	Variable	ESD or EFTR in experienced centers; avoid repeated incomplete snare attempts.
Firm non-lifting lesion with invasive pattern	Depression, ulceration, or deep invasive optical features	High	Oncologic surgery with lymph node assessment.

## Treatment selection algorithm and clinical decision-making

A practical algorithm begins by separating lesions with convincing deep invasion from lesions that appear endoscopically curable. High-definition white-light inspection should be combined with image-enhanced assessment of morphology, vascular and surface pattern, focal depression, lifting behavior, and prior manipulation. Cross-sectional staging is appropriate when advanced disease is suspected. Selective EUS can be considered when uncertainty remains and the result is likely to change management.

For a large low-risk lesion without evidence of submucosal invasion, EMR is generally preferred because it is efficient, widely available, and associated with a low perforation risk. When superficial invasion is suspected or an intact specimen is required to assess depth, lymphovascular invasion, differentiation, budding, and margins, ESD should be considered in an expert center. EFTR is reserved for selected small non-lifting or difficult-location lesions that are not suitable for standard EMR and in which ESD is not the best option. Convincing deep invasion should lead to surgical evaluation rather than progressively more aggressive local endoscopic attempts.

### Post-resection histopathologic risk stratification

**Figure 1:**
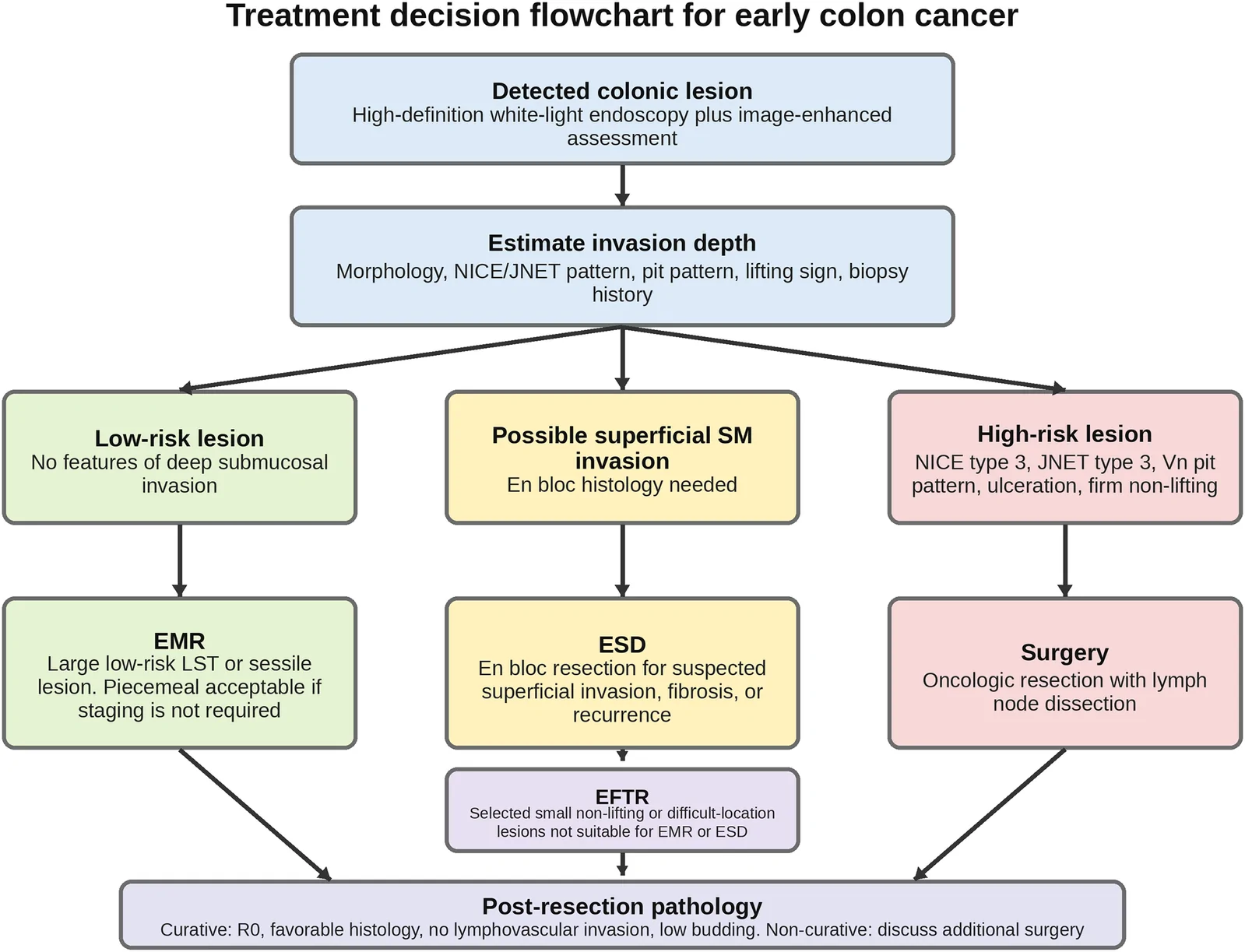
Risk-based treatment decision flowchart for early colon cancer. The algorithm integrates optical diagnosis, estimated invasion depth, lesion lifting and prior manipulation, feasibility of EMR, ESD, or EFTR, and post-resection histopathologic risk. Lesions with convincing deep invasion should be referred for oncologic surgery rather than local endoscopic treatment.

The resection specimen determines whether local therapy is oncologically adequate. Curative assessment should consider *en bloc* and R0 status, histologic differentiation, lymphovascular invasion, tumor budding, and invasion characteristics. Japanese cancer guidelines and other contemporary evidence emphasize that adverse histology, rather than depth alone, is central to estimating nodal risk and deciding on additional surgery (6, 31). Pathology review should be standardized, and borderline cases benefit from multidisciplinary discussion. A risk-based treatment pathway is shown in Figure 1.

The ranges shown in Table 3 are approximate evidence-based summaries rather than pooled estimates. They reflect heterogeneous studies and should be interpreted with the caveats described in the text (11, 12, 20–22, 25, 28, 30).

**Table 3:** Structured quantitative comparison of endoscopic resection techniques for early colon cancer.

Outcome or domain	EMR	ESD	EFTR	Key evidence limitation
Typical role	Most large low-risk nonpedunculated lesions	Lesions requiring *en bloc* staging, including suspected superficial invasion	Selected small non-lifting, fibrotic, recurrent, or difficult-location lesions	Technique selection is strongly confounded by lesion biology and complexity.
*En bloc* resection	Often not feasible when lesions exceed 20 mm; piecemeal common	Approximately 90% or higher in experienced series	High when complete cap capture is achieved	Definitions and lesion size distributions vary.
R0 resection	Limited after piecemeal resection	Approximately 80% to 90% in expert practice	Approximately 70% to 85% in many series	Pathology handling and inclusion of benign versus invasive lesions differ.
Local recurrence	Historically about 10% to 20% after piecemeal EMR; low single digits achievable with modern margin ablation	Usually below 2% after curative *en bloc* resection	Variable; longer-term oncologic data limited	Surveillance intensity and recurrence definitions are heterogeneous.
Perforation	Usually low, roughly 0.5% to 2% in contemporary practice	Commonly about 3% to 8%, strongly expertise dependent	Full-thickness wall capture is intentional; failure of secure closure is the critical concern	Adverse-event reporting and case complexity differ.
Delayed bleeding	Generally a few percent; higher with large right-sided defects and antithrombotic exposure	Generally a few percent	Bleeding occurs but is not the only major safety concern	Risk modification by location and closure strategy is substantial.
Procedure time	Usually shortest	Longest	Short to moderate for suitable lesions	Time depends on lesion size, access, fibrosis, and operator experience.
Oncologic evidence	Strong for benign and selected low-risk lesions; piecemeal T1 resection limits staging	Most mature endoscopic *en bloc* evidence for lesions requiring precise histology	Less mature for early cancer and long-term survival	No valid inference from unadjusted cross-study comparisons.
Training and access	Broadly available	High training burden; benefits depend on volume and referral systems	Device and expertise dependent	Regional infrastructure materially affects outcomes.

## Post-resection management, local recurrence, and surveillance

Post-resection management depends on completeness of excision, *en bloc* versus piecemeal technique, pathology, lesion size, and whether the initial treatment was curative. The first task is to distinguish residual or recurrent mucosal neoplasia, which is often amenable to further endoscopic therapy, from a non-curative cancer resection that carries unresolved nodal risk.

After piecemeal EMR of a lesion 20 mm or larger, early scar assessment is recommended because most residual or recurrent tissue is detected at the first surveillance examination and can frequently be treated endoscopically (23). High-definition and image-enhanced inspection should be used. Repeat EMR, avulsion techniques, or ablation may be appropriate for small residual foci; dense fibrosis or recurrent lesions may require ESD or EFTR in expert centers.

**Figure 2:**
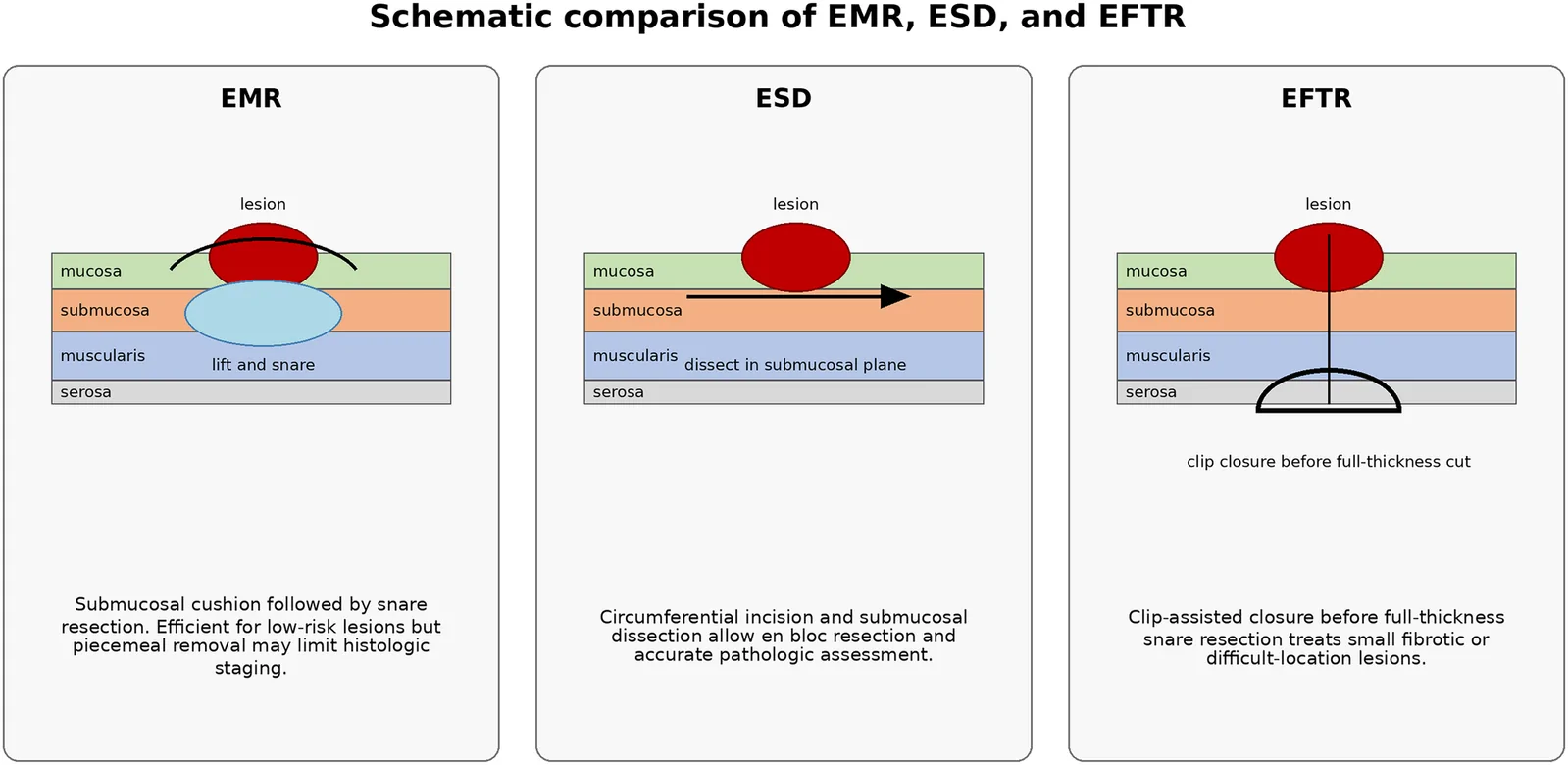
Schematic comparison of EMR, ESD, and EFTR. EMR relies on snare resection after submucosal lifting or selected underwater capture; ESD uses controlled submucosal dissection to achieve *en bloc* resection; and device-assisted EFTR closes the wall before full-thickness resection. The techniques are complementary and should be selected according to invasion risk, need for intact histology, fibrosis, lesion size, location, and local expertise.

After *en bloc* R0 resection of low-risk T1 cancer, surveillance can be individualized according to pathology and background colorectal neoplasia risk. In contrast, high-risk histology or a non-curative resection should prompt multidisciplinary discussion of additional oncologic surgery. Endoscopic surveillance alone does not address lymph node risk. A practical surveillance framework is summarized in Table 4, and the complementary procedural principles of EMR, ESD, and EFTR are illustrated in Figure 2.

**Table 4:** Practical surveillance and local recurrence management after endoscopic treatment.

Clinical situation	Suggested approach	Management focus
Piecemeal EMR of lesion 20 mm or larger	First scar assessment at approximately 3 to 6 months, with subsequent timing guided by findings and guideline context	Careful scar inspection; treat residual tissue; verify complete eradication.
*En bloc* R0 resection with low-risk T1 histology	Structured colonoscopic follow-up, commonly within 6 to 12 months, then individualized	Confirm local control and detect metachronous lesions; no additional surgery if curative criteria are met.
ESD with non-curative or high-risk histology	Endoscopic surveillance alone is insufficient	Multidisciplinary review and consideration of surgery with lymph node dissection.
EFTR for selected non-lifting lesion	Scar and clip-site assessment individualized to pathology and completeness	Assess residual tissue, closure-site changes, and need for additional endoscopic or surgical treatment.
Local residual or recurrent neoplasia	Early reassessment after salvage therapy	Repeat EMR, avulsion, ESD, EFTR, or surgery according to size, fibrosis, location, and invasive risk.

## Comparison of major guideline recommendations

Major European, Japanese, and US recommendations share several core principles: careful optical assessment before resection, avoidance of piecemeal treatment when superficial invasive cancer requires intact histology, referral of complex lesions to appropriately experienced centers, and short-interval surveillance after piecemeal resection of large lesions (11–14, 23). Differences arise primarily in the practical positioning of ESD and in the infrastructure assumed by each guideline.

ESGE recommends EMR as the standard approach for most large nonpedunculated adenomatous lesions and suggests ESD for selected lesions in high-volume centers, particularly when *en bloc* resection is required. JGES provides more detailed and broader operational indications for colorectal ESD, reflecting longstanding expertise and training pathways. USMSTF guidance emphasizes complete endoscopic removal, advanced imaging assessment, referral of lesions not confidently removable by the initial endoscopist, and structured surveillance. These differences should not be interpreted as simple disagreement: they reflect different training environments, access to ESD, referral networks, and healthcare systems. Table 5 summarizes the principal similarities and differences.

**Table 5:** Comparison of major guideline recommendations relevant to large and early malignant colonic lesions.

Domain	ESGE	JGES	US Multi-Society Task Force	Clinical interpretation
Large low-risk nonpedunculated lesions	EMR is standard for most lesions 20 mm or larger	EMR used for lesions suitable for snare resection; ESD considered according to morphology and need for *en bloc* resection	EMR by an endoscopist experienced in advanced polypectomy is emphasized	Broad agreement that EMR is appropriate for most large lesions without invasive features.
Need for *en bloc* histology	En bloc techniques, including ESD, when superficial invasive carcinoma is suspected and standard EMR is inadequate	Detailed ESD indications for lesions difficult to resect *en bloc* by snare or with suspected superficial invasion	En bloc removal favored when cancer staging would be compromised by piecemeal resection	All prioritize intact staging when invasive cancer is plausible.
Position of ESD	Selective, especially in high-volume centers	More established and broadly operationalized within Japanese expertise	Selective and expertise dependent; less widely available	Difference mainly reflects infrastructure, training, and availability.
Non-lifting or previously manipulated lesions	Expert referral; consider advanced *en bloc* techniques according to invasion risk	ESD is a key option when fibrosis is present without clear deep invasion	Avoid repeated failed attempts; refer complex lesions to advanced endoscopists	Prior manipulation can convert a manageable lesion into a fibrotic complex lesion.
Recurrence prevention after piecemeal EMR	Strong support for margin thermal ablation after complete visible hot EMR of large lesions	Technique and surveillance recommendations depend on lesion and resection method	Complete removal and appropriate surveillance emphasized	Modern EMR outcomes depend on defect inspection and recurrence-prevention strategy.
Surveillance after piecemeal large-lesion resection	Early repeat colonoscopy, generally within 3 to 6 months	Short-interval assessment according to resection completeness and pathology	Approximately 6-month surveillance after piecemeal resection of lesions 20 mm or larger	All endorse early scar reassessment; exact timing and wording differ.

## Discussion

### Current controversies and areas of uncertainty

#### EMR versus ESD in Western practice

The central controversy is whether broader adoption of ESD improves patient outcomes enough to justify longer procedures, greater training requirements, and higher perforation risk for lesions that can be managed by high-quality EMR. ESD clearly provides superior *en bloc* and R0 resection, but modern EMR with meticulous defect inspection and margin ablation has narrowed the recurrence disadvantage for low-risk lesions (20, 21, 25). Therefore, low-volume ESD should not automatically be assumed superior to expert EMR for a clearly low-risk granular lesion.

Conversely, using piecemeal EMR for a lesion with a meaningful probability of superficial invasive cancer can destroy the very pathologic information needed to decide whether surgery is necessary. A reasonable Western strategy is centralization: use high-quality EMR for low-risk lesions and refer lesions requiring intact histology, advanced dissection, or management of fibrosis to centers with sufficient ESD volume. This approach acknowledges both oncologic needs and real-world variation in expertise.

#### The role of EFTR

EFTR is sometimes portrayed as a general alternative to ESD or surgery, but current evidence supports a narrower role. Its greatest value is in selected small, non-lifting or fibrotic lesions and difficult locations where complete snare resection is unlikely and ESD is unattractive or unavailable (28, 30). Device cap capacity limits lesion size, appendiceal-orifice treatment carries a distinct appendicitis risk, and long-term oncologic data for early cancer remain limited (29). The unresolved question is not whether EFTR works, but which lesion subgroups derive enough benefit to offset device limitations and site-specific complications.

#### Clinical readiness of artificial intelligence-assisted endoscopy

Randomized trials show that real-time computer-aided detection can increase adenoma detection, including in multicenter screening settings (32, 33). This is the most mature evidence base for endoscopic artificial intelligence. It should not be extrapolated directly to claims that AI improves early colon cancer detection, accurately determines invasion depth across diverse platforms, prevents interval cancer, or improves survival.

Implementation questions remain substantial: external validity across populations and endoscopy systems, false-positive alerts, automation bias, operator dependence, data drift, interoperability, workflow burden, cost, and medicolegal responsibility. Reflecting this uncertainty, the 2025 AGA living guideline did not establish a universal mandate for CADe-assisted colonoscopy and emphasized uncertainty around patient-important long-term outcomes (34). Clinical readiness should therefore be judged by validated use case rather than by the broad label of AI.

### Patient-centered outcomes and shared decision-making

Technique selection has consequences beyond *en bloc* and R0 rates. Patients may value shorter hospitalization, faster recovery, avoidance of colectomy, preservation of bowel anatomy, fewer repeat procedures, lower out-of-pocket costs, or greater certainty that a single operation addresses both the primary lesion and lymph nodes. These preferences can point in different directions. A patient who strongly prioritizes organ preservation may accept intensive surveillance and the possibility of later surgery, whereas another may prefer one-stage oncologic surgery over a staged pathway with residual uncertainty.

Shared decision-making is especially important when more than one strategy is oncologically reasonable. The discussion should include the probability that an endoscopic specimen will prove non-curative, the possibility of additional surgery, procedure-specific adverse events, recovery time, surveillance burden, comorbidity and frailty, anesthesia risk, access to an expert center, travel and time costs, and local operator experience. The existence of an advanced endoscopic technique does not by itself make that technique the patient-preferred option.

A major evidence gap is the relative scarcity of prospective patient-reported outcome and quality-of-life data comparing EMR, ESD, EFTR, and surgery. Future studies should measure symptom burden, time to normal activity, decisional regret, treatment burden, financial toxicity, and quality of life alongside technical and oncologic endpoints.

### Non-resectional endoscopic therapeutic technologies

Curative endoscopic treatment of early colon cancer remains fundamentally resection based because resection provides both local treatment and definitive histopathology. Photodynamic therapy has been studied in colorectal cancer, but the clinical literature is heterogeneous and includes palliative and investigational contexts rather than evidence supporting replacement of complete EMR, ESD, EFTR, or oncologic surgery for resectable early disease (35). Other local drug-delivery or intratumoral approaches should similarly be regarded as investigational until prospective studies demonstrate durable local control, oncologic safety, and patient-centered benefit.

### Future directions and emerging technologies

Future research should prioritize clinically actionable comparisons rather than descriptive device novelty. Prospective multicenter studies are needed to validate integrated optical algorithms that combine morphology, image-enhanced patterns, lifting characteristics, and selective adjunctive imaging. Pragmatic comparative studies should evaluate modern EMR with margin ablation against ESD in well-defined low-risk lesion groups, using recurrence, adverse events, procedure time, cost, need for additional surgery, and patient-reported recovery as co-primary domains.

For EFTR, prospective registries should use central pathology review, clear lesion-size and fibrosis definitions, standardized adverse-event reporting, and long-term follow-up. For ESD, training research should define objective competency metrics rather than relying only on case counts. For AI, external validation, prospective monitoring after deployment, and patient-level outcomes should take precedence over image-level accuracy alone.

Molecular and digital pathology tools may eventually refine nodal-risk estimation after local resection, but they should be evaluated against strong clinical baselines and established histopathologic variables. A new biomarker is useful only if it changes a management decision and improves outcomes beyond current risk stratification.

### Limitations and knowledge gaps

Several limitations affect both the evidence base and this review. First, most comparative evidence is nonrandomized. Lesion morphology, size, fibrosis, suspected invasion, patient fitness, and local expertise strongly influence technique selection, producing selection and referral bias. Second, outcomes are volume dependent. Expert-center ESD or EFTR results may not generalize to low-volume practice, while older Western ESD series may underestimate results achievable after structured training and centralization.

Third, definitions vary. Technical success, R0 resection, curative resection, recurrence, and adverse events are not uniformly reported. Pathology processing and criteria for high-risk T1 disease also differ across studies and regions. Fourth, long-term oncologic evidence is less mature for EFTR than for EMR and ESD, and even apparently favorable survival comparisons between endoscopy and surgery are vulnerable to confounding by indication.

Fifth, the review is a structured narrative synthesis, not a systematic review. The search was targeted rather than exhaustive, formal risk-of-bias tools were not applied study by study, and no *de novo* meta-analysis was performed. Quantitative ranges are intended to aid clinical interpretation, not to create false precision. Finally, patient-reported outcomes, quality of life, treatment burden, and cost-effectiveness remain underrepresented relative to technical endpoints.

## Conclusion

Modern endoscopic management of early colon cancer is best understood as a risk-based treatment continuum rather than a contest among procedures. Accurate optical assessment determines whether local therapy is appropriate. EMR remains the principal option for most large low-risk nonpedunculated lesions; ESD is favored when *en bloc* resection and intact histologic staging are required; EFTR provides a complementary solution for selected small non-lifting, fibrotic, or difficult-location lesions; and surgery remains necessary for convincing deep invasion or non-curative pathology with clinically important nodal risk.

The comparative evidence must be interpreted in context. ESD achieves higher *en bloc* and R0 resection and very low local recurrence after curative resection, but it demands greater expertise and carries higher procedural burden. Contemporary EMR outcomes have improved with margin ablation and structured surveillance. EFTR is promising but constrained by lesion size, anatomy, and less mature long-term oncologic evidence. Regional guideline differences largely reflect the expertise and infrastructure assumed by each system.

The next phase of progress should focus on better comparative evidence, standardized outcomes, centralized training, patient-reported outcomes, and careful validation of AI and other emerging technologies. In everyday practice, the most defensible strategy is multidisciplinary and patient centered: choose the least invasive treatment that still provides adequate oncologic staging and control, while making the possibility of additional surgery, surveillance burden, and local expertise explicit.
